# Supplementary material for: Arabidopsis and maize terminator strength is determined by GC content, polyadenylation motifs and cleavage probability
Source: Nat Commun. 2024 Jul 12;15:5868. doi: 10.1038/s41467-024-50174-7 (PMC11245536; doi:10.1038/s41467-024-50174-7)
Supplement: Supplementary file 1 — Supplementary Information [file 41467_2024_50174_MOESM1_ESM.pdf]

Arabidopsis and Maize Terminator Strength is Determined by GC Content, Polyadenylation Motifs and Cleavage Probability

Supplementary Figures 1-10
Supplementary Tables 1 and 2

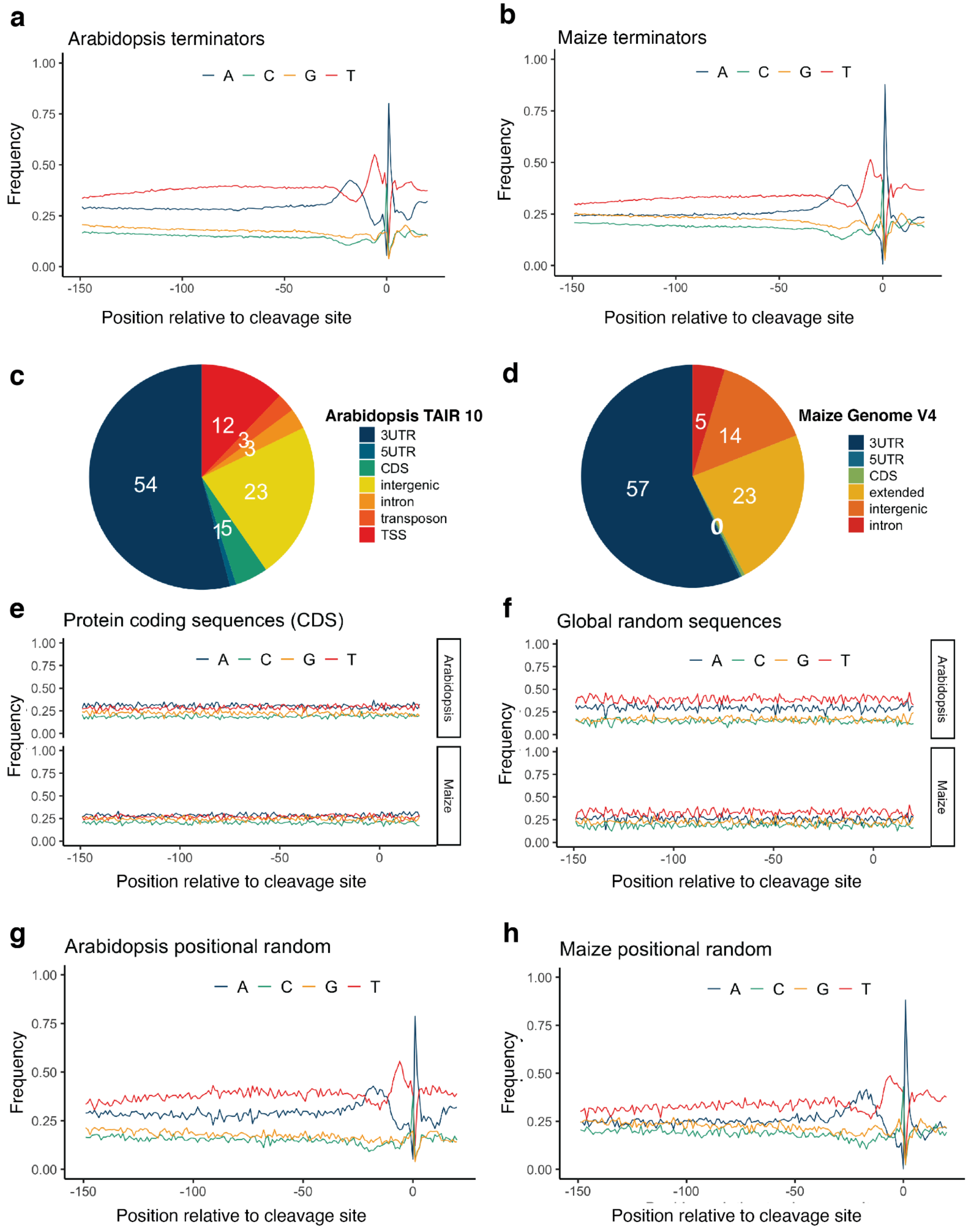

Supplementary Figure 1. **Nucleotide composition for terminators in our library. a, b** Per-position nucleotide frequencies of *Arabidopsis* (**a**) or maize (**b**) terminators. **c, d** Pie charts showing the distribution of the cleavage site location (annotated according to the *Arabidopsis* TAIR10 and the maize B73v4 genome annotations) for the terminators from *Arabidopsis* (**c**) or

maize (**d**) in our library. **e** Per-position nucleotide frequencies for controls derived from coding sequences (CDS) in *Arabidopsis* or maize. **f** Per-position nucleotide frequencies of randomized sequences with an overall (Global random) nucleotide composition similar to average *Arabidopsis* or maize terminator. **g, h** Per-position nucleotide frequencies for randomized sequences with a per-position (Positional random) nucleotide composition similar to an average *Arabidopsis* (**g**) or maize (**h**) terminator.

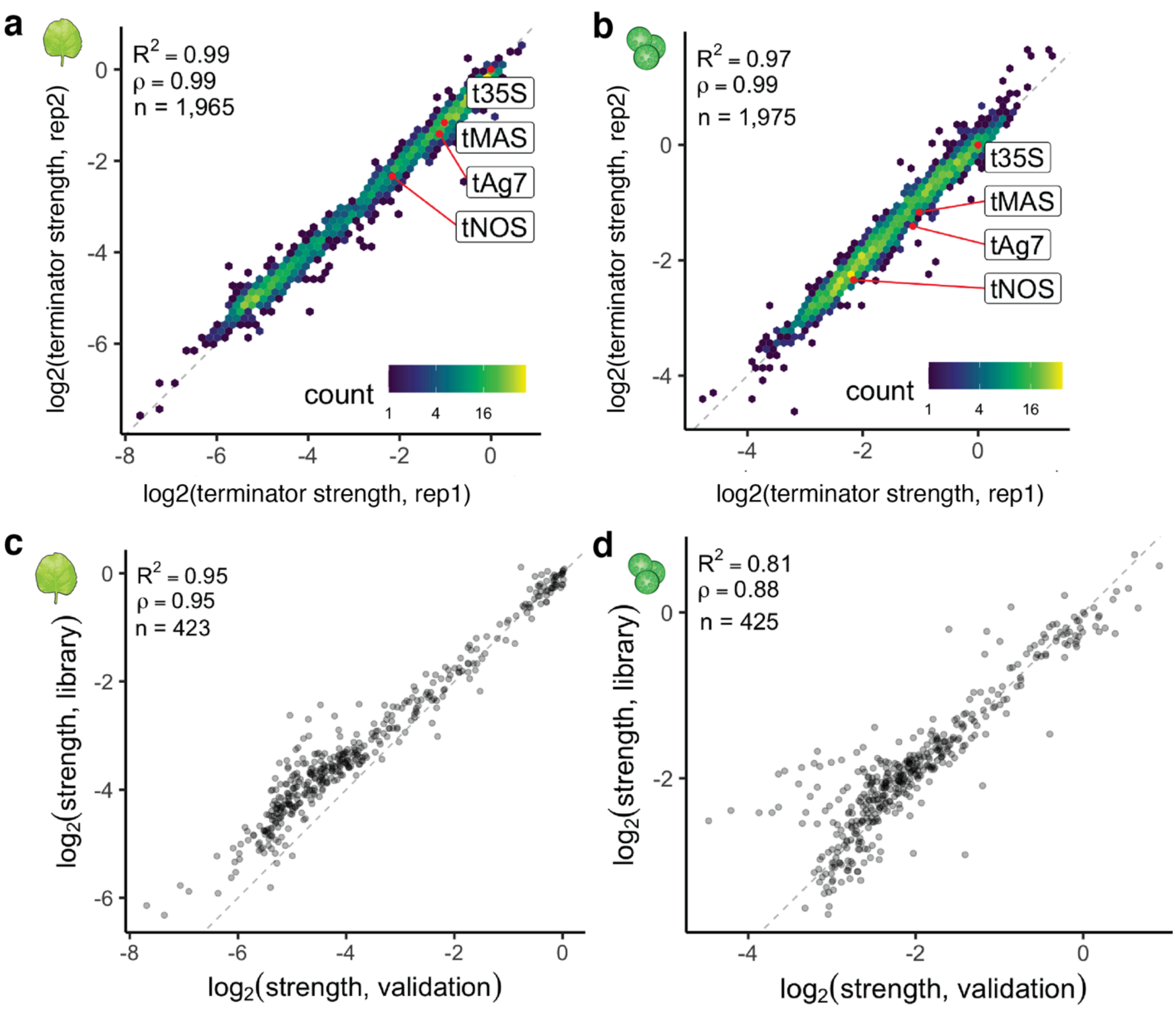

Supplementary Figure 2. **Plant STARR-seq yields highly reproducible results across libraries. a, b** Hexbin plots (as defined in Figure 1.) of the correlation between two biological replicates of Plant STARR-seq with the validation library in tobacco leaves (**a**) or maize protoplasts (**b**). Commonly used terminators are highlighted in red. **c, d** Correlation between terminator strength as measured in the large-scale library and the validation library in tobacco leaves (**c**) or maize protoplasts (**d**). Pearson's R^2, spearman's ρ, and number (n) of terminators are indicated in all plots.

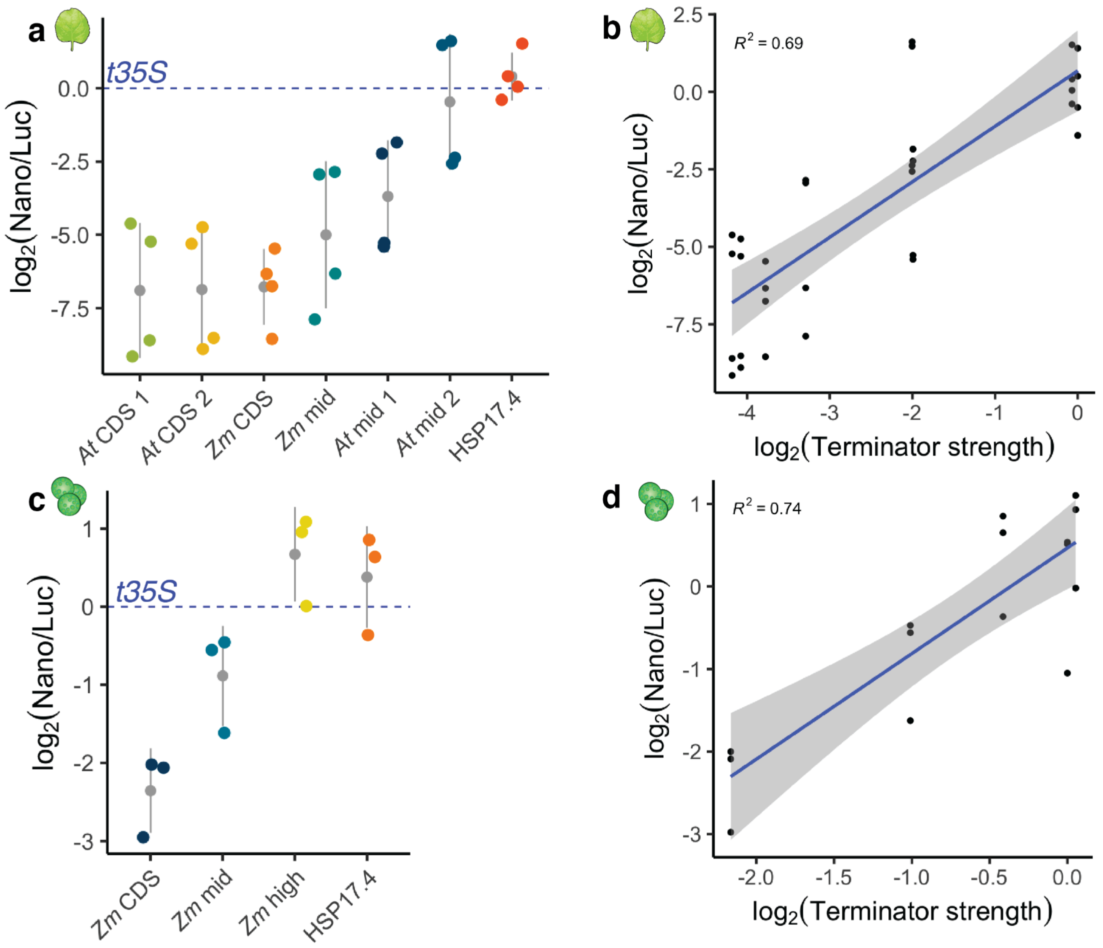

Supplementary Figure 3. **Nanoluciferase activity (protein abundance) reflects terminator strength.** Select weak (CDS), intermediate, and strong terminators are cloned immediately downstream of nanoluciferase. The nanoluciferase/luciferase ratio is normalized to a mean of the construct with the 35S terminator per experiment (35S average; $\log_2$ set to 0, dashed blue line). **a,c** Jitter plot of nanoluciferase activity for selected terminators in tobacco leaves **(a)** and maize protoplasts **(c)**. The gray dot denotes the mean and the gray line denotes the variance. **b,d** Dot plot and Pearson's R^2 between terminator strength and nanoluciferase activity of tested terminators in **(b)** tobacco leaves and **(d)** maize protoplasts. Linear regression line is shown as a blue line, and the gray band around the regression line is the 95% confidence interval. Key: {At mid 1= AT1G26300 ; AT mid 2= AT3G23110; Zm mid = Zm00001d012972_T002; At CDS 1=AT3G22360_CDS; At CDS 2= AT5G07380_CDS; Zm CDS = Zm00001d025717_T001_CDS; Zm high = Zm00001d047961_T001; HSP17.4 = AT3G46230}. Raw values and calculated scores from assay are provided in Supplementary Data 5.

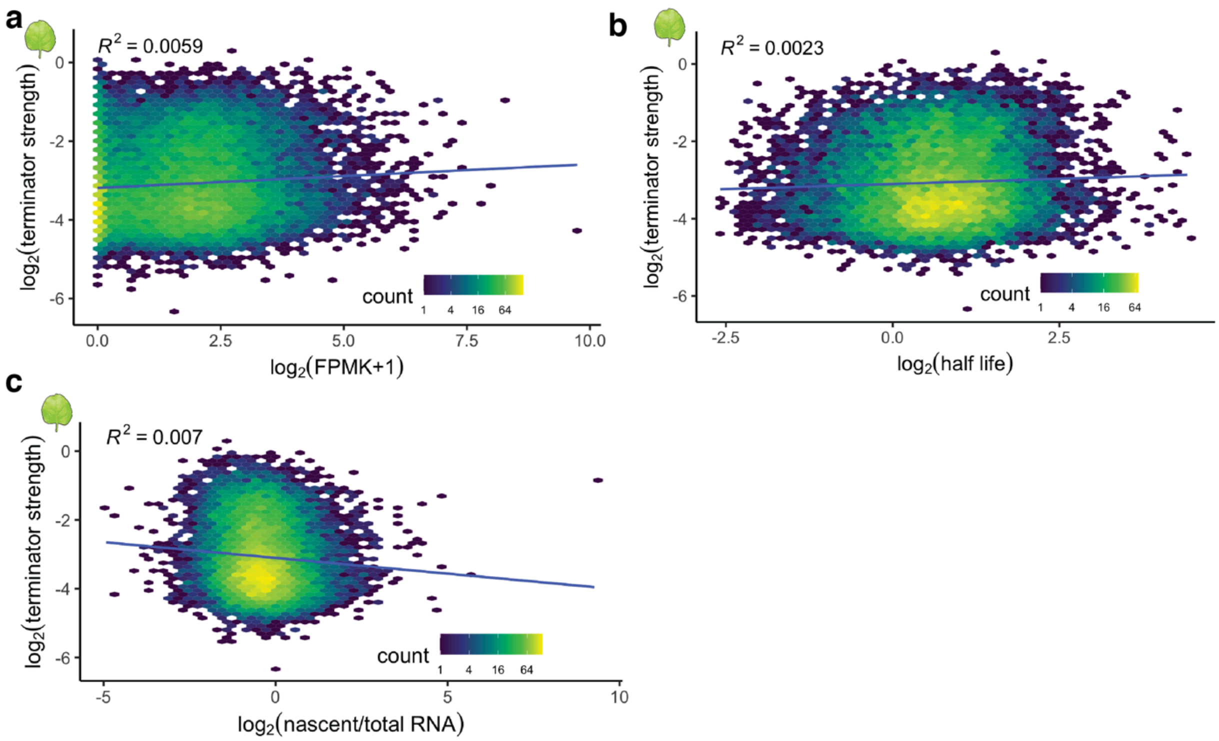

Supplementary Figure 4. **Terminator strength is not correlated to gene expression, mRNA half-life, and nascent transcription. a-c** Hexbin plots (as defined in Figure 1) of the correlation between the strength of *Arabidopsis* terminators and the expression (**a**), mRNA half-life (**b**), or nascent transcription (**c**) of the corresponding genes. Pearson's R^2 is indicated.
See the main text for data sources.

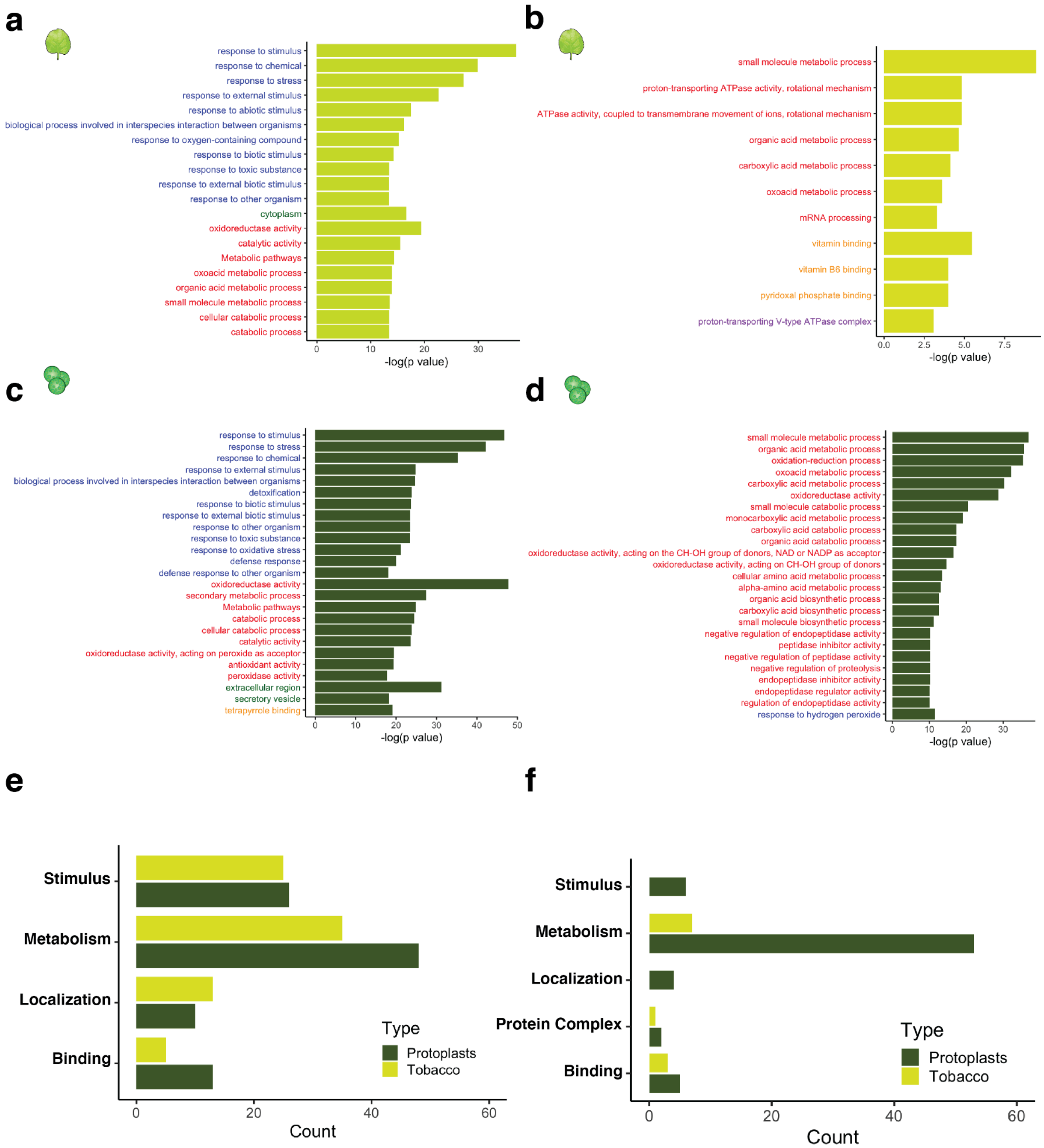

Supplementary Figure 5. **Metabolic and stimulus-responsive genes frequently use strong terminators. a-d** GO terms enriched in genes associated with the top 10% of *Arabidopsis* (**a, c**) or maize (**b, d**) terminators ranked by strength in tobacco leaves (**a, b**) or maize protoplasts (**c, d**). Only the most significant GO terms are shown. The *p* values were determined using the gprofiler2 library in R with gSCS correction for multiple testing. All enriched GO terms and exact *p* values are listed in Supplementary Data 4. **e, f** GO terms for *Arabidopsis* (**e**) or maize (**f**) terminators were collapsed into 5 major categories and counted for each assay system.

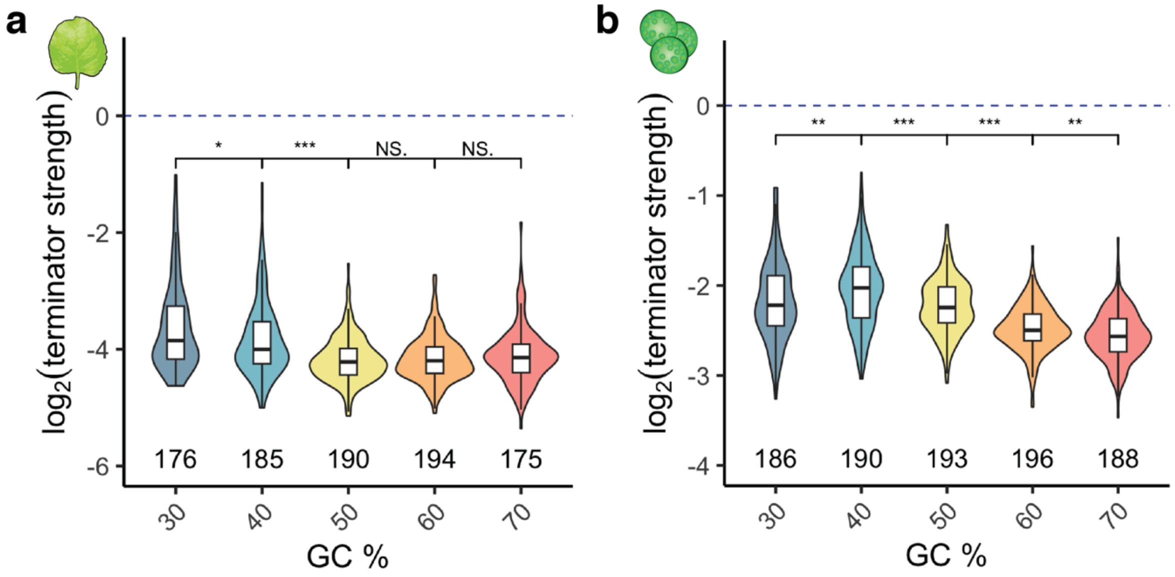

Supplementary Figure 6. **Optimal GC content for terminators is species-specific. a,b** Violin plots, box plots, and significance levels (as defined in Figure 1) of terminator strength in tobacco leaves (**a**) or maize protoplasts (**b**) for randomized sequences with the indicated GC content.

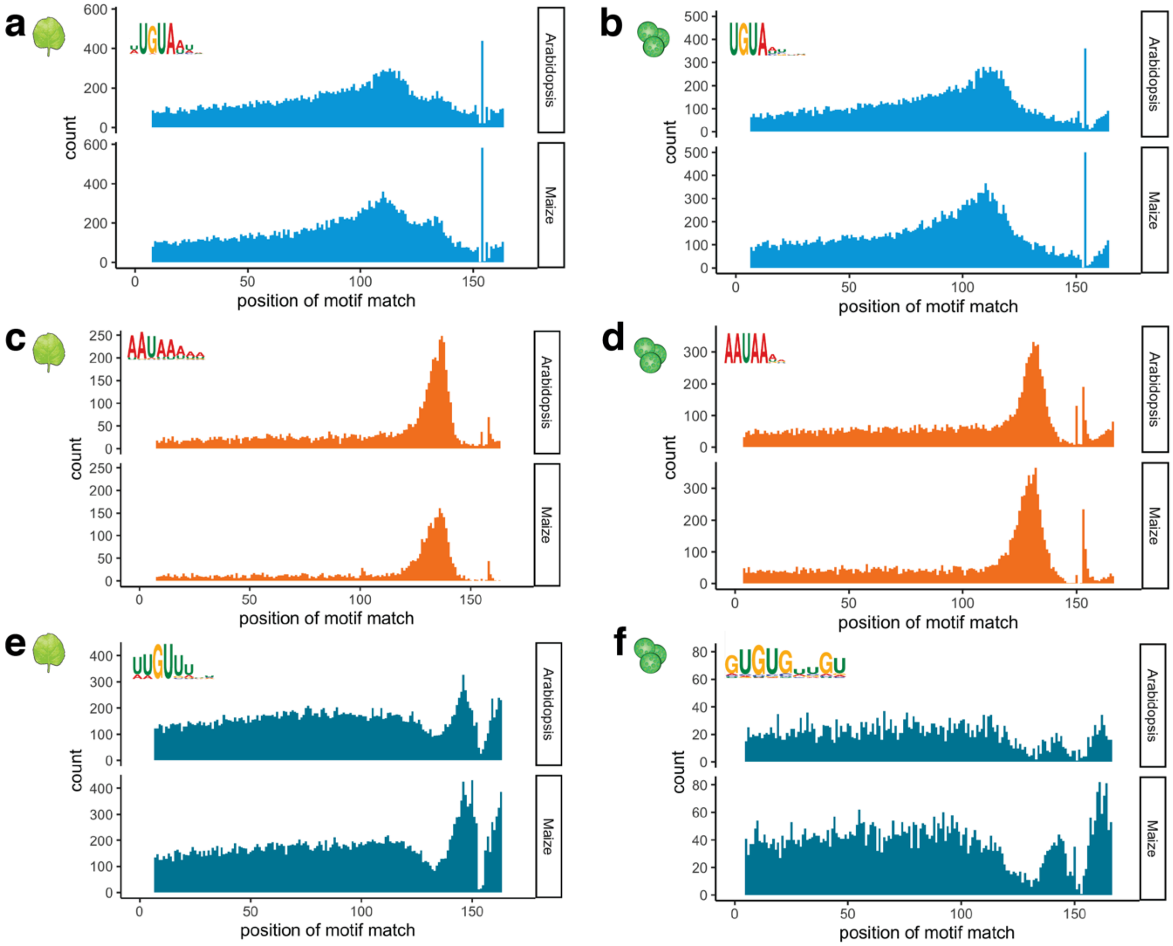

Supplementary Figure 7. **Polyadenylation motifs show distinct localization profiles.**
a-f Histograms showing the number of *Arabidopsis* and maize terminators with a UGUA motif (**a**,**b**), an AAUAAA motif (**c**,**d**), or a U/G-rich motif (**e, f**) at the indicated position. The motifs were discovered in terminators with high strength in tobacco leaves (**a, c, e**) or maize protoplasts (**b, d, f**).

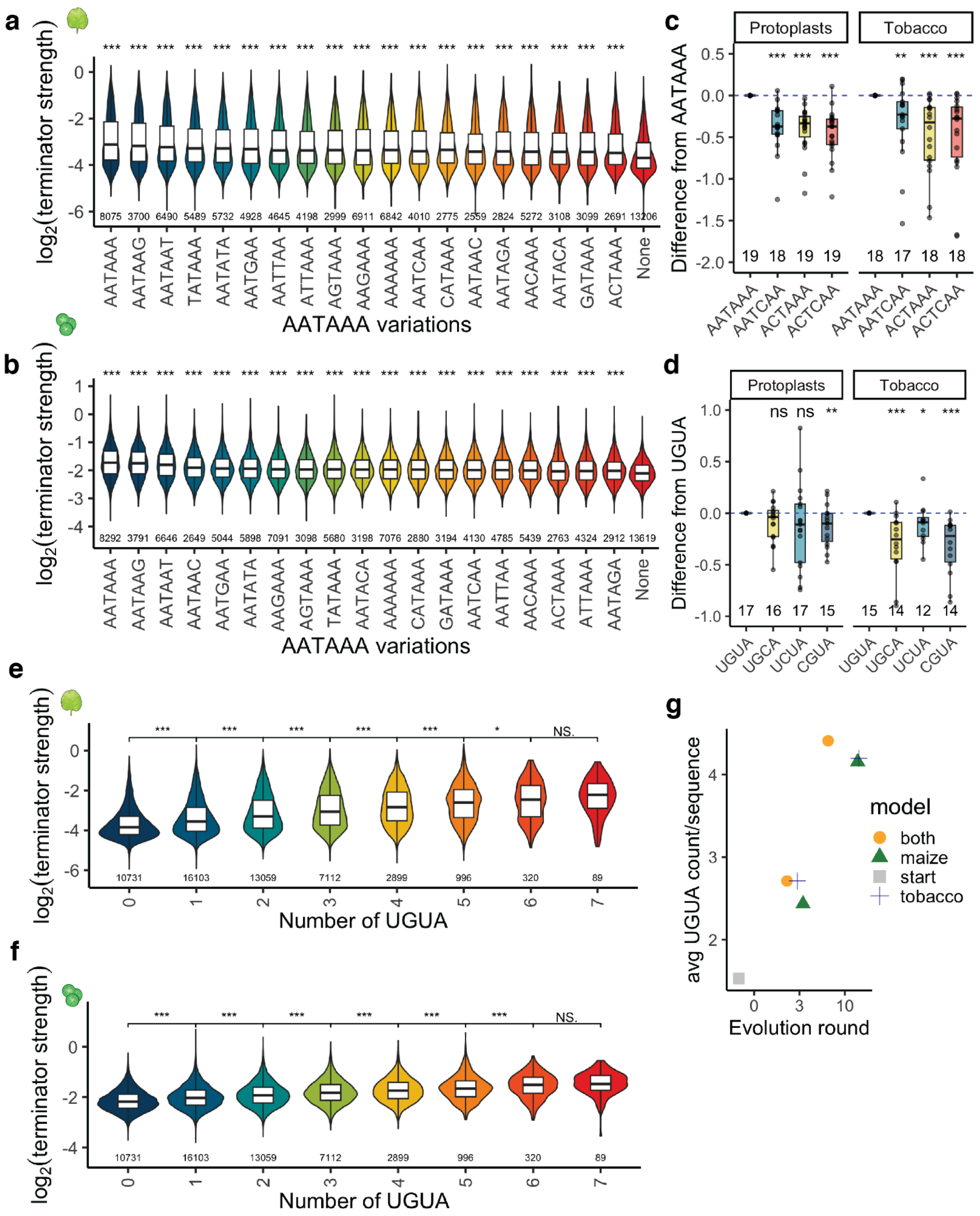

Supplementary Figure 8. **Cleavage and polyadenylation motifs are sensitive to mutations.** **a, b** Violin plots, box plots, and significance levels (as defined in Figure 1) of terminator strength in tobacco leaves (**a**) or maize protoplasts (**b**) for terminators with the indicated variants of the AAUAAA motif. Terminators without any AAUAAA motif variant (None) are also shown. **c, d** Box

plots and significance levels (as defined in Figure 4) of the strength of terminators with the indicated variants of the AAUAAA (**c**) or UGUA (**d**) motif relative to the strength of the corresponding wild type terminator (set to 0). **e-f** Violin plots, box plots, and significance levels (as defined in Figure 1) of terminator strength in **(e)** tobacco leaves and **(f)** maize protoplasts of terminators with varying numbers of UGUA motifs. **g** Jitter plots of the average number of UGUA per terminator through 0, 3, and 10 rounds of *in silico* evolution.

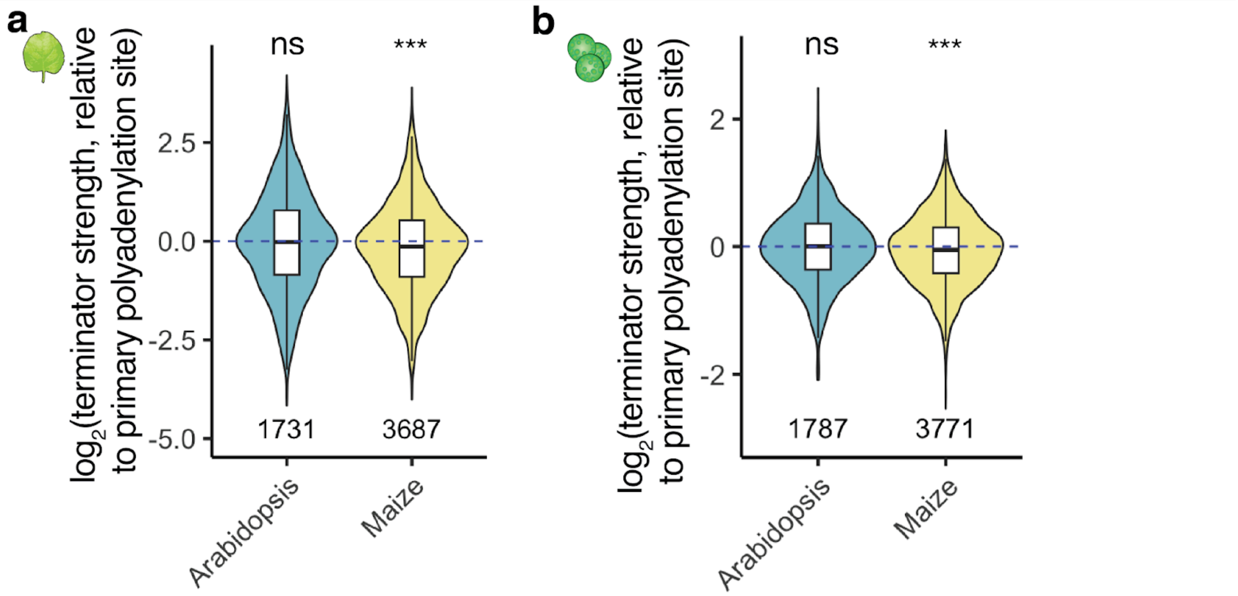

Supplementary Figure 9. **Terminators derived from primary or secondary polyadenylation sites are indistinguishable by terminator strength. a,b** Violin plots, box plots, and significance levels (as defined in Figure 1) of the terminator strength in tobacco leaves (**a**) or maize protoplasts (**b**) of the experimentally determined secondary polyadenylation site of *Arabidopsis* and maize genes relative to the primary polyadenylation site of the same gene (set to 0).

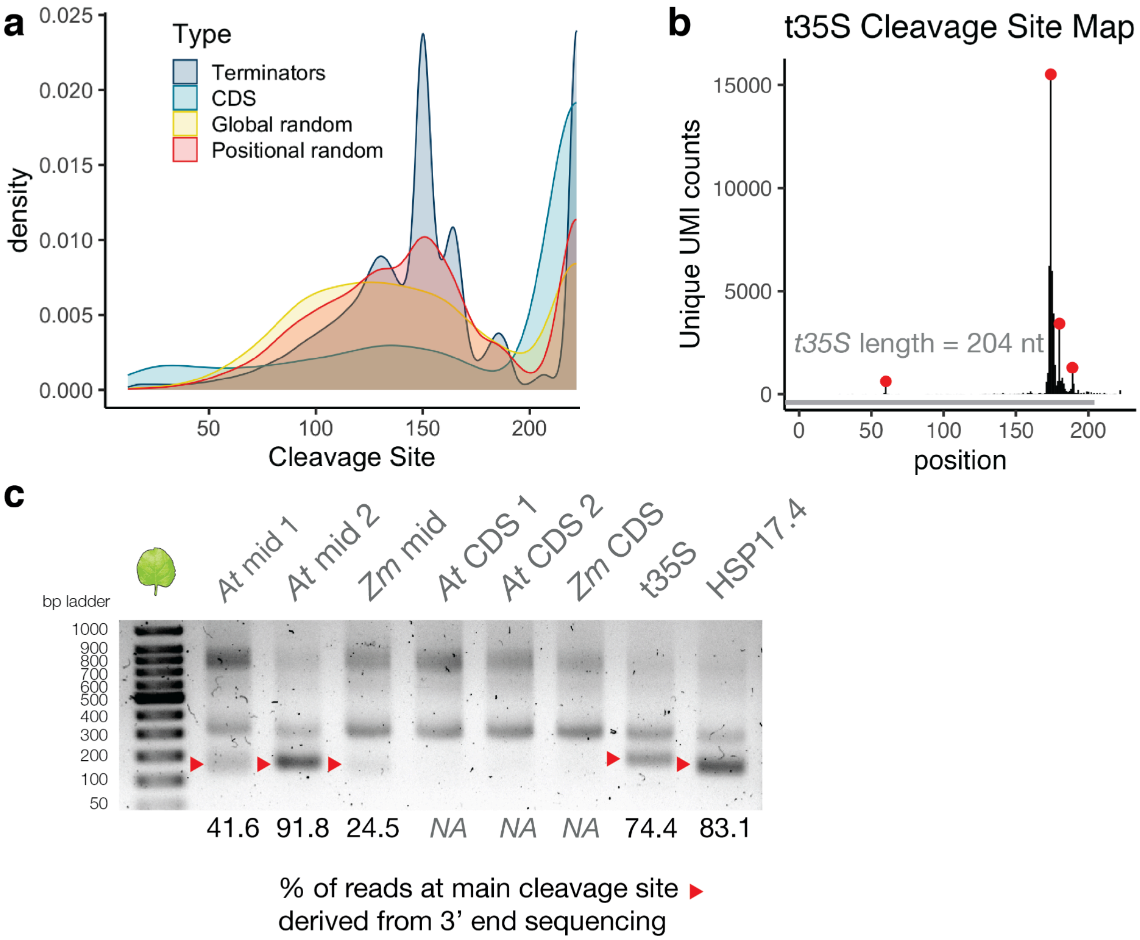

Supplementary Figure 10. **Cleavage site positions differ between *bona fide* terminators and control sequences. a** Kernel density distribution of all cleavage site positions for plant terminators (Terminators), sequences from coding regions (CDS), and randomized sequences with an overall (Global random) or per-position (Positional random) nucleotide frequency similar to an average *Arabidopsis* or maize terminator. **b** Cleavage site map of the CaMV 35S terminator (length=204). **c** RNA was extracted from replicate 1 of the nanoluciferase assay (shown in Supplemental Fig 3A) and reverse transcribed using the same oligo(DT) primer for the 3' end sequencing method. cDNA was amplified and run on a 1.0% agarose gel to resolve cleavage. Red triangles denote the site of primary cleavage determined by 3'end sequencing. Key: {At mid 1= AT1G26300 ; AT mid 2= AT3G23110; Zm mid = Zm00001d012972_T002; At CDS 1=AT3G22360_CDS, At CDS 2= AT5G07380_CDS; Zm CDS = Zm00001d025717_T001_CDS, HSP17.4 = AT3G46230}.

Supplementary Table 1. **Terminator library composition.**

Type	Count	Description
Arabidopsis terminators (main cleavage site)	22,204	Sequences surrounding main polyadenylation and cleavage site of *Arabidopsis* genes (-150 to +20 relative to cleavage site)
Arabidopsis terminators (secondary cleavage site)	2,325	Sequences surrounding secondary polyadenylation and cleavage site (≥30% of total reads) of *Arabidopsis* genes (-150 to +20 relative to cleavage site)
Maize terminators (main cleavage site)	25,685	Sequences surrounding main polyadenylation and cleavage site of maize genes (-150 to +20 relative to cleavage site)
Maize terminators (secondary cleavage site)	4,407	Sequences surrounding secondary polyadenylation and cleavage site (≥30% of total reads) of maize genes (-150 to +20 relative to cleavage site)
CDS	1,178	Coding sequences (170 bp) with similar overall GC content as *Arabidopsis* or maize terminators (589 from each species)
Fixed GC content	1,000	Randomized sequences (170 bp) with a GC content of: 30%, 40%, 50%, 60%, or 70% (200 each)
Global random sequences	400	Randomized sequences (170 bp) with overall nucleotide frequency similar to an average *Arabidopsis* or maize terminator (200 per species)
Positional random terminators	2,000	Randomized sequences (170 bp) with per-position nucleotide frequency similar to an average *Arabidopsis* or maize terminator (1,000 per species)
Commonly used terminators	4	35S, Ag7, NOS, and MAS terminators

Supplementary Table 2. **Primers and DNA fragments used in this study.**

**Sequence**	**Purpose**
GTTCCACTTCTGTATAGGAGCCAATTG	linearize pPSup to build pPSt (fwd)
GGAATTCGATATCAAGCTTATCGATACCG	linearize pPSup to build pPSt (rev)
CGGTATCGATAAGCTTGATATCGAATTCCGCTGCCGTCTTCAGT<br>GCCTGCAGGCATAGAAGACCCGTGACGAGACCACAAGGCGC<br>GCCTAGTGGTCTCCTAAGGGAGACGGGATCCTACAGCTAGCC<br>GTCTCGAGGTGTTCCACTTCTGTATAGGAGCCAATTG	Golden Gate cassettes for pPSt (dsDNA fragment)
GGTCGACGAAGACGGGCTG	amplify 35S promoter + Zm00001d041672 5'UTR (fwd)
GAGAGGAAGACGGTCACNNBNNGNNBNNGNNBNNBCATCGG<br>GGAAGGCTGGG	amplify 35S promoter + Zm00001d041672 5'UTR (rev; contains barcode)
GCGCCGTCTCCTAAG<170bp_terminator>AGGTCGAGACGGTG<br>C	terminator oligo pool
GCGCCGTCTCCTAAG	amplify terminator oligo pool (fwd)
GCACCGTCTCGACCT	amplify terminator oligo pool (rev)
CAGGTCGACGGTCTCCGTGAGCAAGGGCGAGGAG	amplify GFP (fwd)
TCCTCTAGAGGTCTCGCTTACTTGTACAGCTCGTCCATGCC	amplify GFP (rev)
AGTTCAGCCACATGGTCCTGCTGGAGTTC	delete central portion of GFP (fwd)
GGACCATGTGGCTGAACTTGTGGCCGTTTA	delete central portion of GFP (rev)
GGACGACGTCGTCTCCTAAGCTCTAGCTAGAGTCGATCGACAA<br>GCTCGAGTTTCTCCATAATAATGTGTGAGTAGTTCCCAGATAAG<br>GGAATTAGGGTTCCTATAGGGTTTCGCTCATGTGTTGAGCATAT<br>AAGAAACCCTTAGTATGTATTTGTATTTGTAAAATACTTCTATCAA<br>TAAAATTTCTAATTCCTAAAACCAAAATCCAGTACTAAAATCCAG<br>ATAGGTCGAGACGACGTCGTAC	template 35S terminator
GGACGACGTCGTCTCCTAAGCTCTAGCTAGAGTCGATCG	amplify 35S terminator (fwd)
GTACGACGTCGTCTCGACCTATCTGGATTTTAGTACTGGATTTT<br>G	amplify 35S terminator (rev)
GGACGACGTCGTCTCCTAAGGATCGTTCAAACATTTGGC	amplify NOS terminator (fwd)
GTACGACGTCGTCTCGACCTGATCTAGTAACATAGATGACACC	amplify NOS terminator (rev)
GGACGACGTCGTCTCCTAAGCTTGGACTCCCATGTTGGCAAA<br>GGCAACCAAACAAACAATGAATGATCCGCTCCTGCATATGGGG<br>CGGTTTGAGTATTTCAACTGCCATTTGGGCTGAATTGTAGACAT<br>GCTCCTGTCAGAAATTCCGTGATCTTACTCAATATTCAGTAATCT<br>CGGCCAATATCCTAAATGTGCGTGGCTTTATCTGTCTTTGTATT<br>GTTTCATCAATTCATGTAACGTTTGCTTTTCTTATGAATTTTCAAA<br>TAAATTATCAGGTCGAGACGACGTCGTAC	template MAS terminator
GGACGACGTCGTCTCCTAAGCTTGGACTCCCATGTTG	amplify MAS terminator (fwd)
GTACGACGTCGTCTCGACCTGATAATTTATTTGAAAATTCATAAG<br>AA	amplify MAS terminator (rev)

GGACGACGTCGTCTCCTAAGGCTAGCTATATCATCAATTTATGT ATTACACATAATATCGCACTCAGTCTTTCATCTACGGCAATGTAC CAGCTGATATAATCAGTTATTGAAATATTTCTGAATTTAAACTTGC ATCAATAAATTTATGTTTTTGCTTGGACTATAATACCTGACTTGTT ATTTTATCAATAAATATTTAAACTATATTTCTTTCAAGATGGGAATT AGGTCGAGACGACGTCGTAC	template Ag7 terminator
GGACGACGTCGTCTCCTAAGGCTAGCTATATCATCAATTTATG	amplify Ag7 terminator (fwd)
GTACGACGTCGTCTCGACCTAATTCCCATCTTGAAAGAAATATA GT	amplify Ag7 terminator (rev)
GAACTTGTGGCCGTTTACG	reverse transcription for barcode sequencing
AATGATACGGCGACCACCGAGATCTACAC<8bp_index2>CCTCG GCCTCCCTGTCC	amplify barcode for NGS (fwd)
CAAGCAGAAGACGGCATACGAGAT<8bp_index1>CACCCCGGT GAACAGCTCC	amplify barcode for NGS (rev)
CCTCGGCCTCCCTGTCCCCAGCCTTCCCCGATG	NGS read 1 primer for barcode sequencing
CACCCCGGTGAACAGCTCCTCGCCCTTGCTCAC	NGS read 2 primer for barcode sequencing
GTGAGCAAGGGCGAGGAGCTGTTCACCGGGGTG	NGS index 1 primer for barcode sequencing
CATCGGGGAAGGCTGGGGACAGGGAGGCCGAGG	NGS index 2 primer for barcode sequencing
ACGACGCTCTTCCGATCTNNNNNNNNTTTTTTTTTTTTTTTTTTT TTTTTTTTTTTVN	reverse transcription primer for 3’ end sequencing
AATGATACGGCGACCACCGAGATCTACAC<8bp_index2>GGATC ACTCACGGCATGGAC	amplify terminator 3’ end (fwd)
CAAGCAGAAGACGGCATACGAGAT<8bp_index1>ACACTCTTTC CCTACACGACGCTCTTCCGATCT	amplify terminator 3’ end (rev)
GGATCACTCACGGCATGGACGAGCTGTACAAGTAAG	NGS read 1 primer for 3’ end sequencing
Illumina TruSeq read 1 primer (ACACTCTTTCCCTACACGACGCTCTTCCGATCT)	NGS read 2 primer for 3’ end sequencing
Illumina TruSeq index 2 primer (AGATCGGAAGAGCGTCGTGTAGGGAAAGAGTGT)	NGS index 1 primer for 3’ end sequencing
CTTACTTGTACAGCTCGTCCATGCCGTGAGTGATCC	NGS index 2 primer for 3’ end sequencing
AATGATACGGCGACCACCGAGATCTACAC<8bp_index2>CCTCG GCCTCCCTGTCC	amplify terminator library for subassembly (fwd)
CAAGCAGAAGACGGCATACGAGATGCAACCAAGATGCATGTTG TCATCATTGGCTCC	amplify terminator library for subassembly (rev)
GGATCACTCACGGCATGGACGAGCTGTACAAGTAAG	NGS read 1 primer for subassembly sequencing
GATGCATGTTGTCATCATTGGCTCCTATACAGAAGTGGAACACC T	NGS read 2 primer for subassembly sequencing
CCTCGGCCTCCCTGTCCCCAGCCTTCCCCGATG	NGS index 1 primer for subassembly sequencing

CACCCCGGTGAACAGCTCCTCGCCCTTGCTCAC	NGS index 2 primer for subassembly sequencing
GAGTAACCATCAACGGAGTGACC	Amplify nanoluciferase terminators for gel
ACGACGCTCTTCCGATCT	Amplify nanoluciferase terminators for gel
