# Supplementary material for: Arabidopsis and maize terminator strength is determined by GC content, polyadenylation motifs and cleavage probability
Source: Nat Commun. 2024 Jul 12;15:5868. doi: 10.1038/s41467-024-50174-7 (PMC11245536; doi:10.1038/s41467-024-50174-7)
Supplement: Supplementary file 3 — Description of additional supplementary files [file 41467_2024_50174_MOESM3_ESM.pdf]

Description of additional supplementary files

Supplementary Data 1. Terminator assay scores and sequence features

Supplementary Data 2. Validation Terminator assay scores and sequence features

Supplementary Data 3. Exact p values and medians for all comparisons in figures

Supplementary Data 4. Enriched GO terms and exact p values for Figure 2 and Supplementary Figure 5

Supplementary Data 5. Nanoluciferase assay scores for validation terminators

Supplementary Data 6. Major cleavage and polyadenylation sites for terminators derived from 3'end sequencing in tobacco leaves
